# Clinicians, cooks, and cashiers: Examining health equity and the COVID-19 risks to essential workers

**DOI:** 10.1177/0748233720970439

**Published:** 2020-11-26

**Authors:** Jennifer D Roberts, Katherine L Dickinson, Elizabeth Koebele, Lindsay Neuberger, Natalie Banacos, Danielle Blanch-Hartigan, Courtney Welton-Mitchell, Thomas A Birkland

**Affiliations:** 1 Department of Kinesiology, School of Public Health, 1068 University of Maryland, College Park, MD, USA.; 2 Department of Environmental and Occupational Health, 144805 Colorado School of Public Health, University of Colorado Anschutz, Aurora, CO, USA; 3 Department of Political Science, 6851 University of Nevada, Reno, NV, USA; 4 Nicholson School of Communication and Media, 6243 University of Central Florida, Orlando, FL, USA; 5 Department of Natural and Applied Sciences, 8243 Bentley University, Waltham, MA, USA; 6 Department of Public Administration, 6798 NC State University, Raleigh, NC, USA

**Keywords:** COVID-19, essential workers, social determinants of health, health equity

## Abstract

In Spring/Summer 2020, most individuals living in the United States experienced several months of social distancing and stay-at-home orders because of the coronavirus (COVID-19) pandemic. Clinicians, restaurant cooks, cashiers, transit operators, and other essential workers (EWs), however, continued to work outside the home during this time in order to keep others alive and maintain a functioning society. In the United States, EWs are often low-income persons of color who are more likely to face socioeconomic vulnerabilities, systemic racism, and health inequities. To assess the various impacts of COVID-19 on EWs, an online survey was distributed to a representative sample of individuals residing in six states during May/June 2020. The sample included 990 individuals who identified as EWs and 736 nonessential workers (NWs). We assessed differences between EW and NW respondents according to three categories related to health equity and social determinants of health: (1) demographics (e.g. race/ethnicity); (2) COVID-19 exposure risk pathways (e.g. ability to social distance); and (3) COVID-19 risk perceptions (e.g. perceived risk of contracting COVID-19). EWs were more likely to be Black or Hispanic than NWs and also had lower incomes and education levels on average. Unsurprisingly, EWs were substantially more likely to report working outside the home and less likely to report social distancing and wearing masks indoors as compared to NWs. EWs also perceived a slightly greater risk of contracting COVID-19. These findings, which we discuss in the context of persistent structural inequalities, systemic racism, and health inequities within the United States, highlight ways in which COVID-19 exacerbates existing socioeconomic vulnerabilities faced by EWs.

## Introduction

### Essential workers and COVID-19 risks

At the start of the coronavirus (COVID-19) pandemic in the United States, the economy experienced an unparalleled decline as more than 44 million Americans filed for unemployment benefits over the course of 12 weeks (Lambert, 2020). Since March 15, 2020, when the US Centers for Disease Control and Prevention (CDC) issued social distancing recommendations, many individuals lived through several months of full or partial stay-at-home orders (NYT, 2020). While some maintained employment while working from home, millions who were deemed essential workers (EWs) continued to work outside the home, which increased their risk of contracting COVID-19. In order to understand the impacts of COVID-19 on EWs in comparison to nonessential workers (NWs), this research study, conducted by the Risk and Social Policy Working Group (RSPWG), collected data in May/June 2020 from a representative sample of individuals across six states throughout the United States. Differences between EWs and NWs related to three categories of health equity, and social determinants of health were examined: (1) demographics (e.g. race/ethnicity); (2) COVID-19 exposure risk pathways (e.g. working outside the home, social distancing, mask-wearing, using public transportation, leaving home to care for family); and (3) COVID-19 risk perceptions (e.g. perceived risk of contracting, getting ill from, or dying from COVID-19).

Throughout the early months of the pandemic, the term “essential worker” was associated with health-care workers. These workers, who are majority women (75%), have saved lives and been integral to the COVID-19 response, often working under dangerous and exhausting conditions, and with inadequate personal protective equipment (PPE) (McNicholas and Poydock, 2020; Lancet Editorial, 2020). In many states, health-care workers accounted for approximately 20% of the COVID-19 cases, and as of June 2020, nearly 600 health-care workers had died from work-related COVID-19 transmission (KFF, 2020a, 2020b). While these health-care workers have been praised and applauded for their heroic efforts, there was far less recognition for the millions of other EWs who were employed in fields and industries that extend beyond health care. In fact, approximately 70% of EWs in 2019 were not health-care workers (McNicholas and Poydock, 2020). These workers, who often also lacked adequate PPE, included grocery store clerks, public transit operators, hospital custodial staff, delivery workers, food service staff, farm laborers, factory workers, public safety employees, and many others who were designated as “essential” by state government executive orders during the pandemic (McNicholas and Poydock, 2020). The COVID-19 pandemic has underscored the economic and social distinctions between the salaried emergency room physician treating COVID-19 patients, on the one hand, and the hourly waged bus driver transporting hospital employees to work, on the other hand. Although both workers were considered essential and left their homes at great risk to themselves and their families to keep the country running, the bus driver and others like them were largely ignored, undervalued, and they were at a higher risk of contracting COVID-19 (Bhattarai, 2020).

### Essential workers and health equity

“Essential industries” have been defined as “businesses, organizations, and government agencies whose functions are critical to public health, safety, and economic and national security,” while “frontline workers” are “employees within essential industries who must physically show up to their jobs” (Tomer and Kane, 2020). Unfortunately, the historical and contemporary systems of inequity within the United States have magnified the susceptibilities of EWs, and especially frontline workers, during the COVID-19 pandemic. According to the Kaiser Family Foundation’s Tracking Poll conducted on April 15–20, 34% of American adults indicated that they were a designated EW (Kearney and Muñana, 2020). Based on that study, EWs working outside the home were three times more likely to be Black, and approximately 70% did not have a college degree. Overall, people of color have been found to be overrepresented in many frontline industry occupations, such as bus drivers, postal workers, childcare workers, personal care aides, social workers, and nursing assistants, with approximately 41% identifying as Black, Hispanic, Asian American/Pacific Islander, or some classification other than White (Rho et al., 2020). Likewise, many EWs have also been designated as economically vulnerable, as one in three reported living in a household earning less than US$40,000dollars a year, one in seven lacked health insurance, and millions have relied on government assistance programs (Kearney and Muñana, 2020; Roberts and Tehrani, 2020). Based on data from the 2014–2018 American Community Survey, a substantial percentage of women, particularly women of color, have worked in low-wage jobs, including housekeeping cleaners (60%), nursing assistants (50%), and personal care aides (46%), which are jobs that can increase COVID-19 exposure because of the contact with coworkers and the public (Frye, 2020; Roberts and Tehrani, 2020).

**Figure 1.**
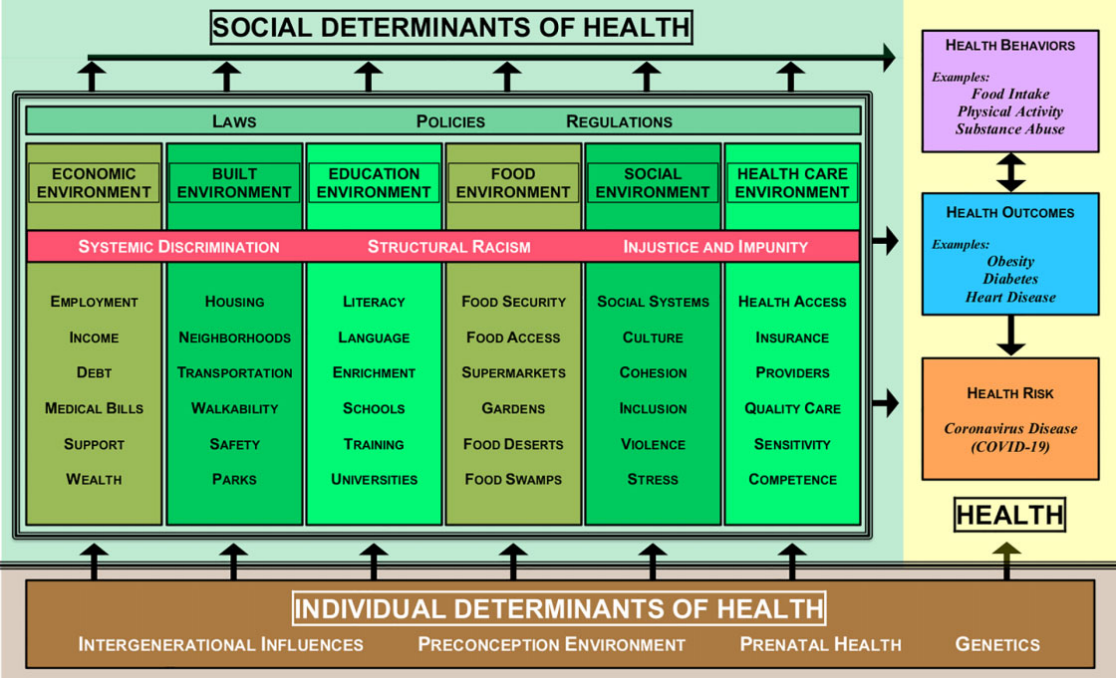
COVID-19 determinants of health model.

As stated by the Robert Wood Johnson Foundation, “health equity means that everyone has a fair and just opportunity to be as healthy as possible. This requires removing obstacles to health such as poverty, discrimination, and their consequences, including powerlessness and lack of access to good jobs with fair pay, quality education and housing, safe environments, and health care” (Braveman et al., 2017). These obstacles have generally been defined as “social determinants of health” or the conditions that negatively or positively impact birth, growth, living, learning, working, playing, and aging (Roberts and Tehrani, 2020). As illustrated in the COVID-19 determinants of health model (Figure 1), an adaptation of the Kaiser Family Foundation model, COVID-19 risk can be attributed to both individual and social determinants of health (Artiga, 2020; Hu and Roberts, 2020). Specifically, a social determinant amalgamation can move along a cascade of harmful circumstances positioned in economic (e.g. low-income employment), built (e.g. overcrowded housing), education (e.g. under-resourced schools), food (e.g. food swamps/deserts), social (e.g. neighborhood violence), and/or health-care (e.g. uninsured) environments. These determinants can negatively impact health through behaviors (e.g. lack of physical activity, smoking) and outcomes (e.g. hypertension, anxiety). According to Garfield et al. (2020), approximately 10% of low-wage workers reported fair or poor health, which can increase the risk of a serious health outcome if one contracts COVID-19. A calamitous example of this occurred in April and May 2020 when there were 15,233 EWs diagnosed with COVID-19 in 239 meat or poultry processing facilities throughout 23 states, ultimately resulting in 86 COVID-19-related deaths (CDC, 2020a). Many of these EWs experienced language and cultural barriers, overcrowded housing, limited transportation options, and many were incentivized to work through sickness (Roberts and Tehrani, 2020), underscoring the role of social determinants of health with regard to COVID-19 exposure risks.

### Risk and Social Policy Working Group Study

In this study, we analyzed the social and economic vulnerabilities faced by EWs, with the idea that these social determinants of health exacerbate existing health disparities and inequities for EWs (Daniel et al., 2018). This research specifically examined demographics, COVID-19 exposure risk pathways, and COVID-19 risk perceptions, comparing EW and NW respondents in order to address the following research questions:

**Research Question A**: Are EWs and NWs significantly different by demographics?
**Research Question B**: Are EWs and NWs significantly different by COVID-19 exposure risks?
**Research Question C**: Are EWs and NWs significantly different by COVID-19 risk perceptions?

## Methods

The RSPWG, an interdisciplinary team of over 15 scholars who came together in response to the COVID-19 pandemic, launched an online survey using a proprietary prearranged pool of respondents recruited by Qualtrics^XM^ (RSPWG, 2020). Data collection took place from May 15 to June 7, 2020, a period during which most states had enacted stay-at-home orders. The survey was distributed to a sample of residents in six states (Massachusetts, Louisiana, Colorado, Iowa, Washington, and Michigan) that was generally representative of the state’s census demographics, as shown in Table 1. The age, race, and income distributions of the sample closely matched each state’s population distributions. Due to difficulties meeting multiple sampling quotas simultaneously, the research team decided to relax the gender quota, and the sample thus included a greater proportion of females than males in all surveyed states. Given the broad similarities between the sample characteristics and population demographics, however, the results can be viewed as representative of the broader population in these six geographically diverse states.

**Table 1.** Characteristics of the survey sample (S) compared to census (C) demographics for six surveyed states.

	All states	Colorado		Iowa		Louisiana		Massachusetts		Michigan		Washington	
Respondents (*n*)	2078	335		312		343		339		401		348	
	s(%)	s(%)	c(%)	s(%)	c(%)	s(%)	c(%)	s(%)	c(%)	s(%)	c(%)	s(%)	c(%)
Gender													
Male	35	35	49	35	50	33	49	39	49	36	49	34	50
Female	64	64	51	65	50	67	51	60	51	63	51	65	50
Age													
18–34	34	36	30	34	27	32	28	34	28	31	26	37	29
35–55	43	41	35	42	32	46	34	44	33	44	33	41	34
55+	23	23	35	24	41	22	38	22	39	25	41	22	37
Race/ethnicity													
Non-Hispanic White	72	69	68	86	85	60	58	74	71	79	75	68	69
Non-Hispanic Black	9	4	5	3	5	30	33	7	8	11	14	5	5
Hispanic/Latino	10	19	22	5	6	4	6	10	12	5	6	14	14
Asian	5	5	3	3	3	3	2	6	7	2	3	9	9
Other/Multiracial	4	4	5	4	4	2	3	3	9	3	5	6	12
Annual household income													
≤US$50,000	41	37	41	48	42	49	52	33	33	40	44	38	33
US$50,001–100,000	32	34	30	32	34	33	27	27	26	32	31	31	31
>US$100,000	28	29	29	20	25	18	21	40	41	28	25	31	36

All respondents reported socioeconomic and demographic data, including race/ethnicity, gender, age, education, income, household makeup, and occupation. Respondents were asked questions on five COVID-19 exposure risk pathways: (1) working outside the home, (2) social distancing, (3) mask-wearing, (4) using public transportation, and (5) leaving home to care for family. These questions were asked as follows: “In the past month, how often did you work outside the home?” (never, 1–3 times/month, weekly, 1+ times/week, everyday); “In the past week, did you keep a social distance from people?” (never, rarely, sometimes, often, always); “In the past week, how often did you wear a face mask indoors?” (never, sometimes, always); “In the past 2 months, how often have you traveled by public transit?” (never, <5 times, 5–10 times, >10 times); “As of today, what are some reasons why you personally may choose or need to leave your home?” (e.g., need to take care of family outside of home). Data on COVID-19 risk perceptions were gathered from questions that asked respondents to report their perceived chances of “getting COVID-19,” “getting seriously ill from COVID-19,” and “dying from COVID-19” on a scale of 0–100%.

In order to identify EWs, all respondents were asked the following question: “Have you been designated as an essential worker by the government or by your employer?” (yes, no, don’t know). Individuals who were unsure of their EW status (*n* = 199) or who were not employed at the time of the survey (*n* = 1134) were not included in the analyses, resulting in a total sample size of 1726 individuals. Individuals were also asked to provide their occupation. Tests of proportions, *χ* ^2^, and analysis of variance were used to determine the differences in demographics, COVID-19 exposure risks, and COVID-19 risk perceptions between EWs and NWs. Statistical analyses were carried out using STATA/MP 16.1.

## Results

### Research Question A: Are EWs and NWs significantly different by demographics?

**Figure 2.**
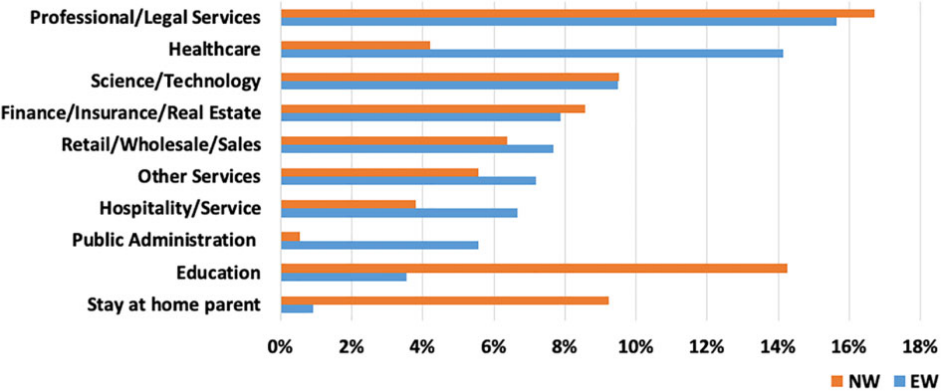
Work and occupations by essential worker status.

Of the 1726 respondents who reported their EW status and were employed at the time of the survey, 990 (57%) were EWs and 736 (43%) were NWs. Work and occupational categories of respondents are presented in Figure 2. Among both EWs and NWs, the largest occupation category was professional and legal services, a broad category encompassing account managers, CEO/CFOs, administrative assistants, attorneys, and managerial positions. Roughly, 16% of EWs and 17% of NWs fell into this category. For EWs, the second most common occupational category was health care: 14% of EWs were employed in this sector, compared to 4% of NWs. Approximately 10% of EWs and NWs were employed in science and technology fields, including engineers and information technology positions. A somewhat larger proportion of EWs were employed in retail/sales (8% EWs vs. 6% NWs), hospitality (7% EWs vs. 4% NWs), and other services (7% EWs vs. 6% NWs). Meanwhile, a much greater proportion of NWs were employed in the education sector (4% EWs vs. 14% NWs). These results confirm that there are key differences between EWs and NWs in terms of their main employment sectors but also that EW status varies within industries and that the EW workforce is highly diverse with respect to occupation.

An examination of the demographics found that EWs and NWs differed significantly in terms of race/ethnicity, income, and education (Table 2). EWs were more likely than NWs to be Black (11.5% vs. 9%) or Hispanic (11.3% vs. 10.3%) and less likely to be Asian (2.5% vs. 5.3%). EWs had lower incomes on average: 25% of EWs made less than US$40,000 and 10% made more than US$150,000, compared to 21% and 19% of NWs, respectively. About 16% of EWs had graduate degrees, compared to 27% of NWs. The EW and NW samples did not differ significantly by gender, age, household size, or number of children.

**Table 2.** All essential vs. non-essential worker demographics.

Parameter	EWs, *n* (% = *n*/990)	NWs, *n* (% = *n*/736)	*χ^2^* (*p* value)
Gender			
Male	409 (41.3)	296 (40.2)	1.0 (0.799)
Female	579 (58.5)	439 (59.7)	
Non-binary	1 (0.1)	0 (0.0)	
Race/ethnicity			
Non-Hispanic White	711 (71.8)	521 (70.8)	15.6 (0.004)
Non-Hispanic Black	114 (11.5)	66 (9.0)	
Hispanic/Latino	112 (11.3)	76 (10.3)	
Asian	25 (2.5)	39 (5.3)	
Other/multiracial	28 (2.8)	34 (4.6)	
Age			
18–34	355 (35.9)	260 (35.3)	0.14 (0.93)
34–55	460 (46.5)	341 (46.3)	
Over 55	175 (17.7)	135 (18.3)	
Annual household income			
≤US$10,000	33 (3.3)	36 (4.9)	37.6 (<0.001)
US$10,001–20,000	62 (6.3)	33 (4.5)	
US$20,001–30,000	76 (7.7)	43 (5.8)	
US$30,001–40,000	74 (7.5)	41 (5.6))	
US$40,001–50,000	69 (7.0)	66 (9.0)	
US$50,001–60,000	85 (8.6)	51 (6.9)	
US$60,001–80,000	132 (13.3)	87 (11.8)	
US$80,001–100,000	120 (12.1)	96 (13.0)	
US$100,001–150,000	235 (23.7)	145 (19.7)	
>US$150,000	104 (10.5)	138 (18.8)	
Education completion			
Some high school	14 (1.4)	11 (1.5)	41.3 (<0.001)
Completed high school	164 (16.6)	105 (14.3)	
Some college	231 (23.3)	133 (18.1)	
2-Year degree	130 (13.1)	58 (7.9)	
4-Year degree	289 (29.2)	230 (31.3)	
Graduate degree	162 (16.4)	199 (27.0)	
Household size			
One person	99 (10.0)	72 (9.8)	6.7 (0.243)
Two people	308 (31.1)	197 (26.8)	
Three people	207 (20.9)	161 (21.9)	
Four people	195 (19.7)	173 (23.5)	
Five people	121 (12.2)	82 (11.1)	
More than six people	60 (6.1)	51 (6.9)	
Children <18 years			
No children	505 (51.0)	351 (60.7)	5.2 (0.26)
One child	206 (20.8)	158 (16.8)	
Two children	179 (18.1)	160 (15.5)	
Three children	74 (7.5)	45 (6.1)	
More than four children	26 (2.6)	22 (3.0)	

EW: essential worker; NW: nonessential worker.

### Research Question B: Are EWs and NWs significantly different by COVID-19 exposure risks?

**Figure 3.**
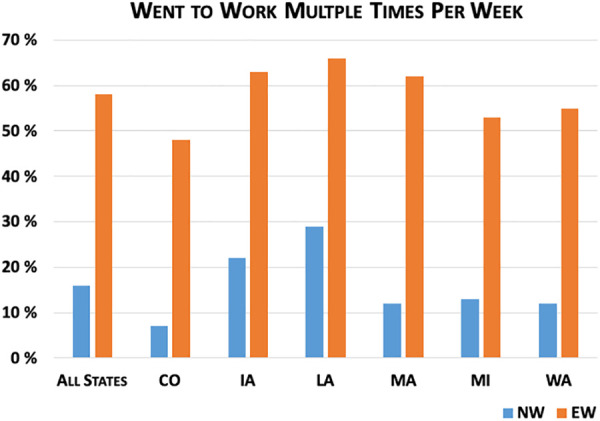
COVID-19 exposure risks (work location) by essential worker status.

**Figure 4. fig4-0748233720970439:**
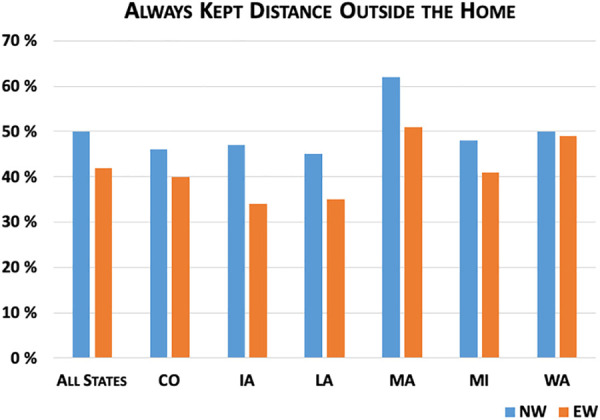
COVID-19 exposure risks (social distance) by essential worker status.

**Figure 5. fig5-0748233720970439:**
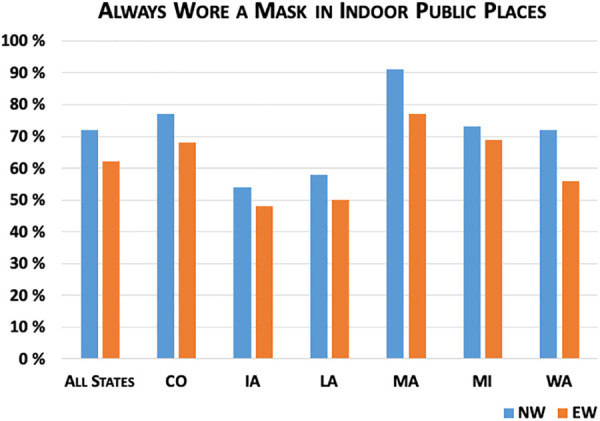
COVID-19 exposure risks (mask-wearing) by essential worker status.

**Figure 6. fig6-0748233720970439:**
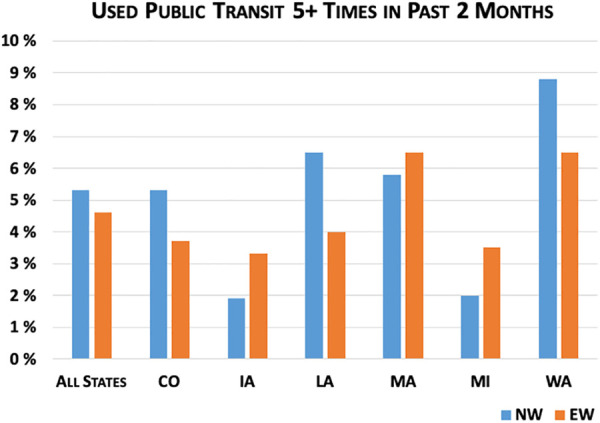
COVID-19 exposure risks (public transit) by essential worker status.

Figures 3 [Fig fig4-0748233720970439] [Fig fig5-0748233720970439] [Fig fig6-0748233720970439] to 7 show differences between EWs and NWs for five COVID-19 exposure risks, with results for the whole sample as well as within each state. Not surprisingly, we found large and statistically significant differences in likelihood of working outside the home between EWs and NWs. Overall, just 16% of NWs reported working outside the home multiple times per week over the study month, compared to 58% of EWs. Rates of working outside the home and differences between EWs and NWs also varied considerably across states. For example, in Louisiana, nearly 30% of NWs and 66% of EWs regularly worked outside the home, compared to 12% of NWs and 53% of EWs in Massachusetts.

**Figure 7.**
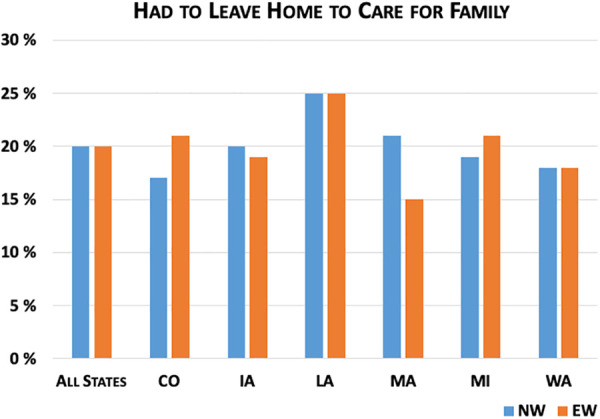
COVID-19 exposure risks (family care) by essential worker status.

We also observed significant differences between the groups in two key risk-reduction behaviors: social distancing and wearing masks. In both cases, EWs were less likely to report engaging in these behaviors than NWs, presumably with a much greater risk than NWs due to the length of time and extent of contact with others while working. Overall, 50% of NWs and 42% of EWs reported that they always kept distance from other people outside their household (*p* value = 0.001). Again we found variation across states, with the highest rates of social distancing reported by both groups in Massachusetts. NW–EW differences were statistically significant at the 10% level in three states (Iowa, Louisiana, and Massachusetts). Follow-up survey questions asked respondents why they may not have been able to keep distance from others at all times outside the home. Nearly half (49%) of EWs indicated that it was not possible for them to keep distance at work, compared to just 12% of NWs (*p* value < 0.001). Fewer individuals (9% of EWs and 7% of NWs) reported that they did not always keep distance because they did not think the risk of COVID-19 was serious (*p* value = 0.06), and a similar proportion of EWs and NWs (about 12%) said that they simply forgot.

For mask-wearing, 72% of NWs reported that they always wore masks in indoor public spaces, compared to 62% of EWs. At the state level, we found significant differences at the 10% or lower level in Colorado, Massachusetts, and Washington. Mask-wearing rates were lowest in Iowa, where less than half (48%) of EWs reported always wearing masks indoors compared to 54% of NWs, and highest in Massachusetts (77% of EWs vs. 91% of NWs). Again, follow-up questions assessed potential barriers to mask-wearing. Less than 4% of EWs and NWs said that they did not own masks. More frequently, 36% of EWs and 31% of NWs said it was hard to breathe in their masks (*p* value = 0.02) and 15% of EWs and 14% of NWs said their masks were uncomfortable (*p* value = 0.18). Roughly 10% of both EWs and NWs said they forgot to wear masks.

We did not find significant differences in the proportion of EWs versus NWs reporting taking public transit more than five times in the past 2 months. These proportions were below 10% across all groups, although they were somewhat higher in Washington (9% of NWs vs. 6.5% of EWs) compared to other states. We also did not observe large differences across EWs and NWs in reported need to leave home to care for family.

### Research Question C: Are EWs and NWs significantly different by COVID-19 risk perceptions?

**Figure 8.**
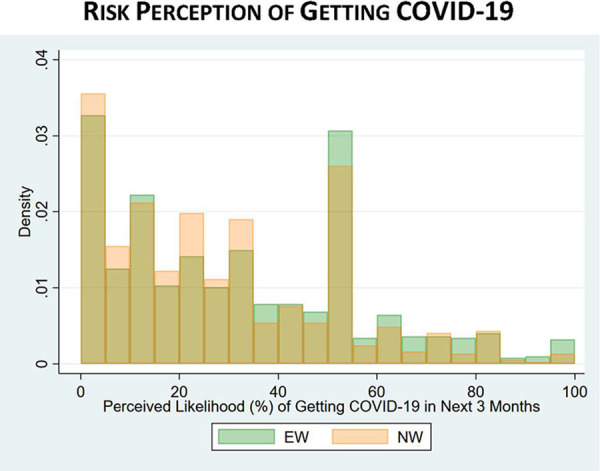
COVID-19 risk perceptions (getting COVID-19) by essential worker status.

**Figure 9. fig9-0748233720970439:**
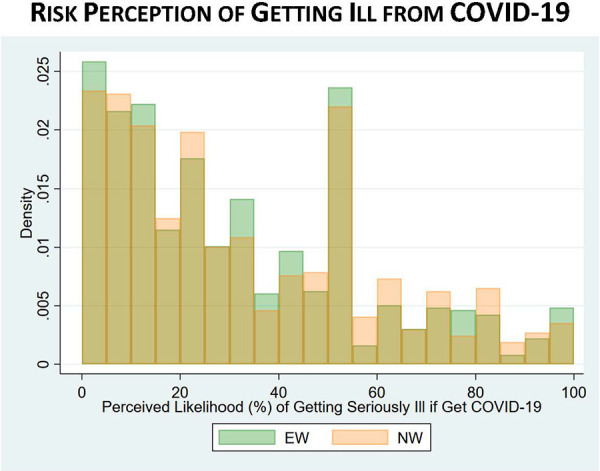
COVID-19 risk perceptions (getting ill from COVID-19) by essential worker status.

With respect to COVID-19 risk perceptions, the difference between EWs and NWs in perceptions of “getting COVID-19” was statistically significant, with EWs perceiving a 31% chance that they would get COVID-19 in the next 3 months compared to 27% among NWs (*p* value < 0.001). However, EWs had similar perceptions of getting seriously ill (31% EWs vs. 32% NWs) or dying (18% EWs vs. 19% NWs) if they got COVID-19. Figures 8 [Fig fig9-0748233720970439] to 10 show histograms of risk perceptions across EW and NW groups. We also asked respondents if they thought they had actually contracted COVID-19: 12% of EWs said yes, compared to 10% of NWs (*p* = 0.16).

**Figure 10.**
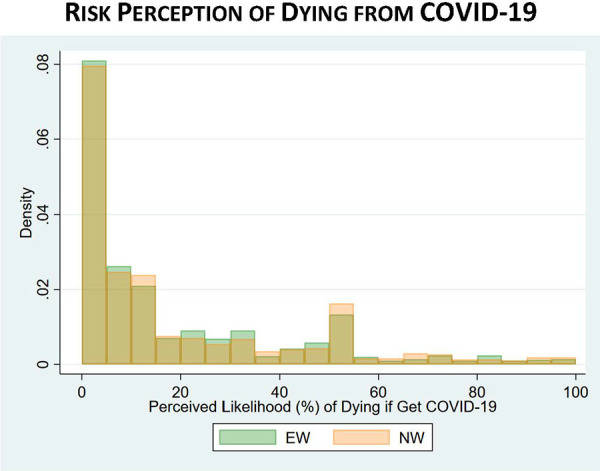
COVID-19 risk perceptions (dying from COVID-19) by essential worker status.

## Discussion

Results from this representative survey of six US states suggest that EWs were more likely to be lower income, persons of color, and have lower levels of education than NWs. Not surprisingly, EWs were more likely to be employed in health-care fields than NWs; however, we also found that many EWs were employed in service professions. Also, as expected, EWs were significantly more likely to report regularly going to work outside the home during the pandemic in comparison to NWs, thus situating them in more frequent contact with other people and increasing their risk of contracting COVID-19. Despite this higher risk of exposure, EWs were less likely to report that they always kept a distance from others and always wore masks, two key protective behaviors that can reduce disease transmission. Many EWs indicated that keeping distance at work was not possible. EWs also perceived a greater personal risk of contracting COVID-19. In this section, we discuss these findings in the context of other research on sociodemographic predictors related to differential COVID-19 exposures and outcomes. Guided by direct quotes from EWs or research experts, we highlight how these differences are tied to and amplified by structural inequalities, systemic racism, and health inequities.

### “Experience has taught all of us that if you’re poor, if you’re of color, you’re going to get services second”

Our study found that there were more Black and Hispanic individuals in the EW category than in the NW category and that the EW category was made up of a diversity of occupations, from those working in professional and legal services to those working in hospitals and grocery stores. This finding resonates with other research reporting that Black and Hispanic workers were overrepresented in transportation, accommodation and food, construction, public administration, and health-care occupations (McCormack et al., 2020). While these industries are necessary for the health, well-being, and the continued operation of the country, research has found a clear link between direct customer service work, COVID-19 infection rates, and COVID-19 racial health disparities (Pryor and Tomaskovic-Devy, 2020). Specifically, both race and EW status have predicted exposure to COVID-19 at work and contributed to the disparate COVID-19 incidence and mortality rates for Black, Hispanic, and other communities of color (Oppel et al., 2020; Pryor and Tomaskovic-Devy, 2020). As of October 28, 2020, Black Americans (108.4 deaths/100,000) have experienced the highest COVID-19 mortality rates compared to Indigenous (90 deaths/100,000), Pacific Islander (68.9 deaths/100,000), Hispanic (73.5 deaths/100,000), White (54.4 deaths/100,000), and Asian (45.4 deaths/100,000) Americans (APM, 2020). When adjusted for age, the gaps in COVID-19 mortality expand even more by race: Black, Indigenous, and Hispanic communities have mortality rates 3.2, 3.1, and 3.2 times that of the White community (APM, 2020).

Pointedly, the disparate mortality rates within the Black community from COVID-19 reflect a complex intersection of social determinants as illustrated in Figure 1, as well as preexisting health conditions attributable to individual and/or social determinants of health. Among the eight preexisting health conditions that the CDC listed as “at increased risk” for severe COVID-19 illness, there have been well documented higher or worse morbidity or mortality rates for seven of the conditions within the Black community, including some cancers, chronic kidney disease, chronic obstructive pulmonary disease, obesity, heart disease, sickle cell disease, and type Ⅱ diabetes (Assari, 2016; Carnethon et al., 2017; CDC, 2020b; DHHS, 2019, 2020; Fuller-Thomson et al., 2016; Lee et al., 2019; NCI, 2019). Yet, even with these health disparities, it is imperative to recognize that institutional and structural inequities influence individual health. As an exemplification of the cascading social determinant effects, Black Americans as well as other communities of color tend to live in food, transit or recreational deserts and more polluted and densely impoverished areas, all of which are related to many if not all of the aforementioned preexisting health conditions (Roberts and Tehrani, 2020). Moreover, it has been found that over 30% of EWs would have to borrow money to pay for an unexpected US$500 medical bill (Kearney and Muñana, 2020).

Compounding these economic circumstances, many Black Americans and other people of color have experienced challenges securing health-care access and insurance (Roberts and Tehrani, 2020). For instance, COVID-19 testing inequities have been observed throughout the pandemic (Tribune, 2020). Some physicians were less likely to refer Black patients for COVID-19 testing when they arrived with recognizable signs of infection, such as cough and fever (Farmer, 2020; Livingston, 2020). Specifically, pilot data revealed that, in comparison to White patients, Black patients with COVID-19 symptoms were six times less likely to receive testing or treatment (Mtshali, 2020). Dismissing Black health complaints and prioritizing patients based on race or income—whether during or outside of the COVID-19 pandemic—further institutionalizes racist structures within the US health-care system that perpetuate vulnerability and health inequity. As best put by Dr George Benjamin of the American Public Health Association, “Experience has taught all of us that if you’re poor, if you’re of color, you’re going to get services second” (Farmer, 2020). Taken together, this information demonstrates how sociodemographic factors such as employment, income, race/ethnicity, along with other determinants of health, interact to decide who lives and who dies (Roberts and Tehrani, 2020).

### “Employees within essential industries who must physically show up to their jobs”

Findings from this study clearly demonstrated a significant difference in likelihood of working outside the home between EWs (58%) and NWs (16%). The most common occupational fields among workers in this RSPWG sample were fairly consistent with other findings on the common employment areas of essential and frontline workers (Tomer and Kane, 2020). Frontline workers, defined as “employees within essential industries who must physically show up to their jobs,” are particularly likely to face a heightened level of risk to COVID-19 exposure as these workers may be in close physical proximity to customers, colleagues, commuters, and/or anyone outside of their household at a much higher frequency (Tomer and Kane, 2020), whereas others deemed EW may have the opportunity to continue working from home or in a space with much lower contact with others. Moreover, compared to all US workers, three-quarters of all frontline workers earn lower than average wages. For instance, the average hourly wage for a cashier is US$11.17, which is below the American livable wage of US$16.54 per hour (Nadeau, 2020; Tomer and Kane, 2020). The obligatory nature of frontline work during the pandemic likely exacerbated these types of preexisting socioeconomic vulnerabilities for many individuals.

### “The part that makes me feel unsafe is the customers”

Innumerable essential and frontline workers have expressed feeling unsafe in workplace settings especially as a result of limited or inadequate PPE, such as face masks or shields (Hammonds et al., 2020). These expressed unsafe work conditions were demonstrated by Occupational Safety and Health data from April 20 to August 20, 2020, which found that the cumulative number of COVID-19 related workplace safety complaints increased over 350% (O’Donnell, 2020). Interestingly, we found that EWs were less able to keep a social distance at work (51%) and wear a mask indoors (62%) than NWs, behaviors that can sometimes be traced directly to the nature of their work (e.g. not being able to keep space from other individuals). Similarly, mask-wearing mandates by employers have varied by state and over time, which not only puts EWs at a greater physical health risk but may also induce an unnecessary level of mental stress and anxiety for EWs when dealing with maskless customers or having to “police” mask-wearing. For instance, as of September 19, 2020, there was no statewide order requiring a face mask in Iowa, unlike the other five states in the RSPWG sample, which helps to explain why mask-wearing rates were lowest in Iowa in our study (Roberts and Mitroff, 2020). In a study conducted by the University of Massachusetts Amherst Labor Center and Center for Employment Equity, one worker explained, “The part that makes me feel unsafe is the customers” (Hammonds and Kerrissey, 2020). Some employers have minimized risk for their EWs by providing employee masks, hand sanitizer stations, 6-feet distance floor signs, instituting mandatory temperature checks for employees, and requiring customers to wear masks upon entry (Sugar, 2020). However, the stringency of these efforts has varied in enforcement, efficacy, and decree.

Another overlooked pathway of COVID-19 exposure for EWs is public transit use for both the riders and the transit operators (Transit Center, 2020). While this RSPWG sample did not find differences in the proportion of EWs and NWs reporting public transit use of more than five times in the past 2 months, our analysis among all transit users did identify a higher percentage of EWs (64%) in comparison to NWs (36%) who used public transit more than 10 times in the past 2 months. This is not surprising given the demographic makeup of EWs in our study. Others have found that Black (34%) and Hispanic (27%) individuals were more likely to use public transit daily or weekly compared to White (14%) individuals, in part because Black (9.5%) and Hispanic (7%) workers are more likely to lack access to a personal vehicle than are White (2.8%) workers (Anderson, 2016; Austin, 2017). Furthermore, Black and Hispanic workers reported using public transit for commuting to work at higher rates than White workers and tended to live further from their place of employment as a result of residential segregation, urban sprawl, transit deserts, and other transportation-related inequities (Anderson, 2016; Bullard, 2006; Harms, 2014).

Moreover, a portion of the EWs in this study worked in the transportation industry, which could magnify COVID-19 risk, as captured in other existing studies. For example, Levenson (2020) tells the story of Jason Hargrove, a city bus driver in Detroit, Michigan, who posted a Facebook video expressing his frustration: “We out here as public workers doing our job trying to make an honest living to take care of our families. But for you to get on the bus and stand on the bus and cough several times without covering up your mouth and you know that we are in the middle of a pandemic, that lets me know that some folks don’t care.” Hargrove died 11 days later from COVID-19 (Levenson, 2020).

## Conclusion

As highlighted in our data and the other studies cited above, EWs and especially those on the front lines have carried an undue burden to protect the health and well-being of others and support the continued operation of the country during the COVID-19 pandemic while simultaneously facing inequitable barriers to minimizing their own risk. This research has confirmed the socioeconomic vulnerabilities faced by EWs, a group made up of a higher proportion of low-income, Black, Hispanic, and other persons of color compared to NWs. Acknowledging these unique vulnerabilities for these groups, including a history of structural racism, discriminatory practices, and the pathologizing of race and poverty, not only reframes our understanding of the COVID-19 disparate impacts but also highlights important considerations for achieving the goal of giving working communities “a fair and just opportunity to be as healthy as possible,” even after the COVID-19 pandemic has run its course.
